# *Lycium barbarum* glycopeptide and luteolin synergistically protect mouse photoreceptors against N-nitroso-N-methylurea-induced degeneration

**DOI:** 10.4103/NRR.NRR-D-24-01473

**Published:** 2025-08-13

**Authors:** Xiu Han, Qihang Kong, Yajing Liu, Xuesong Mi, Shibo Tang, Kwok-Fai So, Ying Xu

**Affiliations:** 1 Guangdong-Hongkong-Macau Institute of CNS Regeneration, Key Laboratory of CNS Regeneration (Jinan University)-Ministry of Education, Guangdong Key Laboratory of Non-human Primate Research, Jinan University, Guangzhou, Guangdong Province, China; 2 Aier Eye Hospital, Jinan University, Guangzhou, Guangdong Province, China; 3 Department of Ophthalmology, The First Affiliated Hospital of Jinan University, Guangzhou, Guangdong Province, China; 4 Aier Academician Station, Changsha, Hunan Province, China; 5 State Key Laboratory of Brain and Cognitive Sciences, Hong Kong Special Administrative Region, China; 6 Co-Innovation Center of Neuroregeneration, Nantong University, Nantong, Jiangsu Province, China

**Keywords:** inflammation, inherited retinal diseases, luteolin, *Lycium barbarum* glycopeptide, N-nitroso-N-methylurea, photoreceptor, reactive gliosis, retinal degeneration, retinitis pigmentosa, wolfberry extract

## Abstract

Photoreceptor degeneration is a major cause of vision impairment in retinal diseases, for which no effective treatment currently exists. Previous research by our team demonstrated that *Lycium barbarum* glycopeptide and luteolin can independently promote photoreceptor survival and function in degenerated mouse retinas, although with limited efficacy. This study evaluated whether a combination of *Lycium barbarum* glycopeptide and luteolin provides enhanced therapeutic benefits compared with either compound alone. Wild-type mice received a daily oral gavage of *Lycium barbarum* glycopeptide and luteolin for 7 days prior to intraperitoneal injection of N-nitroso-N-methylurea to induce photoreceptor damage. The treatment continued for an additional week after injury. Retinal structure and function were subsequently assessed using electroretinogram recordings, visual behavior testing, and immunostaining. Western blot analysis was conducted to investigate the underlying protective mechanisms. The results showed that the *Lycium barbarum* glycopeptide-luteolin mixture significantly increased photoreceptor survival, improved retinal light response, and enhanced visual behavior. Importantly, the combination outperformed either compound alone in protective efficacy. Mechanistic analysis indicated that the mixture suppressed retinal inflammation and modulated the extracellular signal-regulated kinase and Bcl-2-associated X protein/B-cell lymphoma 2 signaling pathways. These findings suggest that the combination of *Lycium barbarum* glycopeptide and luteolin represents a promising therapeutic strategy for photoreceptor degeneration.

## Graphical abstract

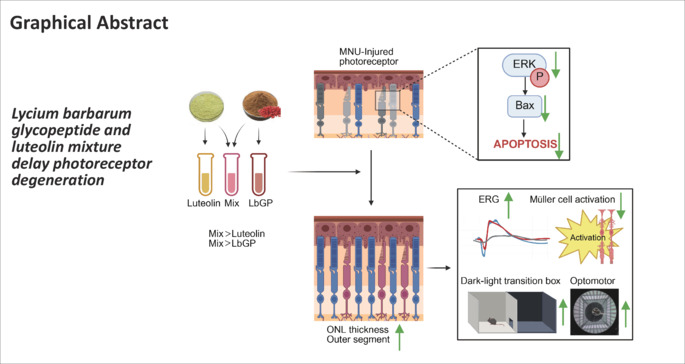

## Introduction

Retinitis pigmentosa (RP) comprises a group of inherited disorders characterized by progressive degeneration of the retina’s light-sensitive cells, leading to gradual vision loss and, in severe cases, complete blindness. It affects approximately 1 in 4000 individuals worldwide, substantially impacting physical and emotional well-being, daily functioning, and mobility (O’Neal et al., 2024). Current treatment strategies for RP include gene therapy, stem cell therapy, nutritional supplementation, neuroprotective agents, hyperbaric oxygen therapy, and retinal transplantation (Nguyen et al., 2023; Confalonieri et al., 2024). Concerns regarding safety, limited efficacy, and high cost have hindered the development of universally effective therapies (Singh et al., 2020; Hirami et al., 2023). Within this context, traditional Chinese medicine (TCM) offers a promising alternative approach involving a comprehensive therapeutic effect and non-invasive delivery. TCM has demonstrated robust efficacy in the treatment of various retinal disorders and has substantially improved patients’ quality of life (An et al., 2020; Qi et al., 2023a).

*Lycium barbarum* (i.e., wolfberry) is a widely recognized TCM agent with protective effects on the eye and liver. Its water-soluble and insoluble extracts have shown therapeutic benefits in animal models of age-related macular degeneration, diabetic retinopathy, glaucoma, and RP (Mi et al., 2012; Liu et al., 2018, 2021a; Lakshmanan et al., 2023). *Lycium barbarum* glycopeptide (LbGP), a major water-soluble component, has a known protective role in the retina and potential therapeutic applications in various conditions (e.g., retinal ischemia–reperfusion injury and glaucoma) (Lakshmanan et al., 2024; Wu et al., 2024). LbGP exerts its effects through mechanisms such as antioxidant, anti-inflammatory, and antiapoptotic pathways (Dai et al., 2023; Kong et al., 2024). Our previous study demonstrated that LbGP significantly improved the survival and function of degenerated photoreceptors in RP models. However, its therapeutic efficacy was limited, likely owing to insufficient anti-inflammatory activity; LbGP only partially suppressed microglia activation (while it significantly reduced the number of Iba-1^+^ cells to 50% of the N-nitroso-N-methylurea (MNU) group, it failed to reduce the number of CD68^+^ cells) and had no effects on Müller cell activation (with 1.50 fold increase of GFAP intensity compared to the control, which was similar to the 1.7 fold increase in MNU group). The addition of a potent anti-inflammatory agent in conjunction with LbGP may enhance its protective effect on the RP retina (Kong et al., 2024).

Luteolin is a flavonoid found in various plants, known for its strong antioxidant properties and ability to inhibit microglia activation (Williams et al., 2004; Dirscherl et al., 2010). In a previous study using the rd10 mouse model, which genetically replicates RP, luteolin effectively suppressed the activation of Müller cells and microglia in the retina. It also reduced the levels of several inflammatory cytokines, including tumor necrosis factor-α and interleukin-1β (Zhang et al., 2022a). On the basis of these findings, we hypothesized that the combination of LbGP with luteolin could enhance the anti-inflammatory capacity of LbGP and improve its protective effect against photoreceptor degeneration.

This study investigated whether the therapeutic efficacy of the LbGP–luteolin mixture exceeds that of either agent alone. MNU, an alkylating toxin, was used to chemically induce rapid photoreceptor-specific degeneration in mice (Tao et al., 2015; Kong et al., 2024). Mice treated with MNU received the LbGP–luteolin mixture; outcomes were compared with those of mice receiving LbGP or luteolin alone. Recovery of visual function and preservation of retinal structure were subsequently assessed.

## Methods

### Animals

C57BL/6J male mice (specific pathogen-free, approximately 21 g) were obtained from Liaoning Changsheng Biotechnology Co., Ltd., Benxi, Liaoning Province, China (License No. SCXK (Liao) 2020-0001). All animals were 7 weeks old at the start of the experiment. Mice were housed under standard laboratory conditions with a controlled 12-hour light/dark cycle and unrestricted access to food and water (humidity: 40%–65%; temperature: 18–23°C). A maximum of five mice were housed per cage to ensure sufficient space. Throughout the experiment, 3R principles (reduction, replacement, and refinement) were strictly followed to minimize animal suffering and injury. The animal experiments were approved by the Laboratory Animal Ethics Committee of Jinan University on March 1, 2022 (approval No. IACUC-20220301-08-02) and conducted in accordance with the National Institutes of Health Guide for the Care and Use of Laboratory Animals (8^th^ ed., National Research Council, 2011).

### Research approach and drug treatment strategies

**Figure 1:**
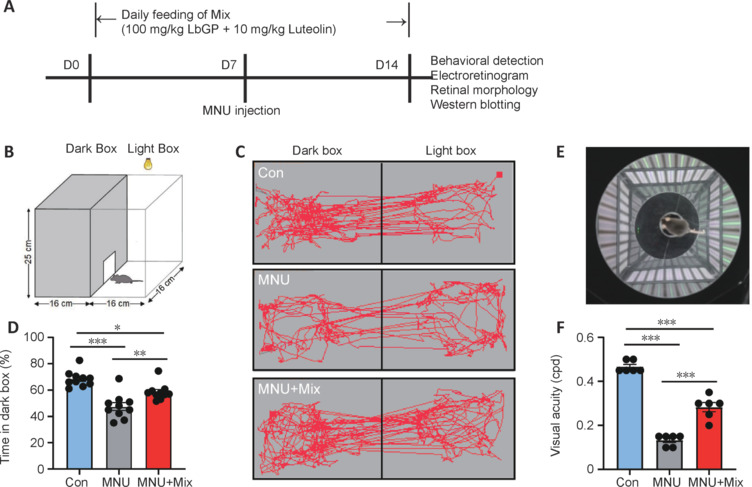
LbGP and luteolin mixture improves visual function in MNU-injured mice. (A) Experimental setup. (B) Schematic of the dark–light transition test. (C) Representative movement patterns of mice in the dark–light box for each group. (D) Percentage of total test time spent in the dark compartment (*n* = 10 mice per group). (E) Schematic of the optomotor system. (F) Visual acuity, expressed in cycles per degree (cpd), for each group (*n* = 6 mice per group). Treatment with the LbGP and luteolin mixture increased the time spent in the dark compartment and improved visual acuity in MNU-injured mice. Data are expressed as mean ± SEM; *P < 0.05, **P < 0.01, ***P < 0.001 (one-way analysis of variance with Tukey’s *post hoc* test). Group: Con, Control; MNU, MNU-induced retinal injury in mice; MNU + Mix, MNU-injured mice treated with a mixture of LbGP and luteolin. D: Day; LbGP: *Lycium barbarum* glycopeptide; MNU: N-nitroso-N-methylurea.

Animals were randomly assigned to three groups: a normal control group of untreated mice (Con, *n* = 44), an MNU-injured group (MNU, *n* = 44), and an MNU-injured group treated with a combination of LbGP and luteolin (LbGP and luteolin mixture, *n* = 47). Animals were randomly allocated to groups by hand as we used before (Kong et al., 2024). LbGP powder (purity ≥ 92.0%), procured from Tianren Goji Biotechnology Co., Ltd. (Zhongwei, China) was dissolved in 0.1% phosphate-buffered saline (PBS). Based on our previous work, a daily dose of 100 mg/kg body weight was selected for its optimal protective effect on the MNU-injured retina (Kong et al., 2024). Luteolin powder (purity ≥ 98.0%), obtained from Mansite Biotechnology Co., Ltd. (Chengdu, China), was first dissolved in 1 N NaOH and diluted with 0.1% PBS; the pH was then adjusted to 7.2–7.4 using 1 N HCl (Liu et al., 2021b). Our previous study using up to 120 mg/kg luteolin in rd10 mice identified 100 mg/kg as the most effective dose. However, considering the potential synergistic effects of co-administration and the risk of adverse effects at high doses, the luteolin dose was reduced to 10 mg/kg. This dosage has been shown to retain anti-inflammatory and antioxidant properties (Zhang et al., 2021). From day 1 to day 14, mice received daily oral gavage of a mixture containing 100 mg/kg LbGP and 10 mg/kg luteolin. On day 7, a single intraperitoneal injection of 40 mg/kg MNU (Macklin Biochemical Technology Co., Ltd, Shanghai, China) was administered to induce photoreceptor-specific degeneration, as described in prior studies (Emoto et al., 2016; Kong et al., 2024). On day 15, visual behavior was evaluated. On day 16, electroretinography (ERG) was performed. Mice were anesthetized by intraperitoneal injection of 1.25% tribromoethanol solution (Merck KGaA, Darmstadt, Germany) at 0.02 mL/g. A schematic overview of the experimental procedures is presented in **Figure 1A**.

In a separate set of experiments, animals were treated with either the mixture, LbGP, or luteolin at the highest combined dose (110 mg/kg). Animals were randomly assigned to three groups: an MNU-injured group treated with LbGP alone (LbGP, *n* = 9), an MNU-injured group treated with luteolin alone (Luteolin, *n* = 9), and an MNU-injured group treated with a combination of LbGP and luteolin (LbGP and luteolin mixture, *n* = 9). Immunostaining was used to compare protective effects on retinal architecture.

### Visual behavioral tests

On day 15 of the study, visual behavior was assessed using a dark–light transition chamber and a custom-designed optomotor apparatus. The dark–light transition chamber, which evaluates the innate preference of mice for darkness over illuminated environments, was used to assess luminance perception, in accordance with a previously described method (Liu et al., 2021b). Each mouse was monitored for 5 minutes, during which its movements were recorded. EthoVision XT 8.0 software (Noldus, Wageningen, the Netherlands) automatically calculated the proportion of time spent in the dark compartment relative to the total test duration.

Visual acuity was evaluated using the optomotor test by observing head movements in response to the rotation of vertical stripes (Chen et al., 2024). A detailed protocol is available in our previously published work (Kong et al., 2024). The spatial frequencies of gratings ranged from 0.1 to 0.5 cycles per degree, rotating at a constant speed of 12 degrees per second. Mice instinctively tracked the moving gratings by turning their heads, a behavior considered an optomotor response. Head movements were video-recorded, and the highest spatial frequency that elicited a visual motor response was manually recorded to determine the visual acuity of each mouse.

### Electroretinography

To assess the response of retinal neurons to light stimulation, mice were kept in complete darkness overnight after the behavioral test. On day 16, ERG was performed using the RETI-scan system (Gaush Meditech Ltd., Beijing, China), in accordance with the protocol described in our previous work (Zhang et al., 2022b). Mice were exposed to full-field green light flashes at increasing intensities ranging from 0.01 to 3.0 cd·s/m^2^. Subsequently, they were light-adapted for 5 minutes using a background illumination of 20.0 cd·s/m^2^. Photopic responses to green flashes at intensities of 3.0 cd·s/m^2^ and 10.0 cd·s/m^2^ were then recorded. RETI-scan software was used to analyze a-wave and b-wave amplitudes, as well as corresponding peak times. For each animal, data from the eye exhibiting the strongest response were selected for analysis.

### Tissue processing

After behavioral tests, mice remained under anesthesia. Cervical dislocation was immediately performed to euthanize the animals. Eyes were promptly enucleated and fixed with 4% paraformaldehyde at room temperature (25°C) for 30 minutes. The eyes were then washed three times with 0.1% PBS; each rinse lasted 5 minutes. An overnight dehydration step in 30% sucrose solution followed. After dehydration, eyes were embedded in optimal cutting temperature compound and rapidly frozen at –40°C. Retinas were longitudinally sectioned through the optic disk at a thickness of 14 µm using a Thermo NX50 cryostat (Thermo Fisher Scientific, Waltham, MA, USA). Sections were then mounted onto glass slides for subsequent analysis.

### Immunofluorescence staining

For immunohistochemical staining, retinal sections were washed three times with PBS containing 0.3% Triton X-100 (Servicebio, Wuhan, China). Sections were then incubated for 2 hours in a blocking buffer consisting of PBS, 0.3% Triton X-100, 5% donkey serum, and 3% bovine serum albumin. Blocked sections were incubated overnight at 4°C with primary antibodies diluted in the same buffer. After thorough washing, sections were incubated with secondary antibodies for 2 hours at room temperature (25°C). After a final series of washes, sections were stained with 4′,6-diamidino-2-phenylindole (DAPI; 1:1000, Electron Microscopy Sciences, Hatfield, PA, USA, Cat# 28718-91-4, RRID: AB_2307445) for 5 minutes to visualize cell nuclei, then mounted onto glass slides and coverslipped. Details of the primary and secondary antibodies are listed in **Table 1**.

**Table 1:** Antibodies used for immunofluorescent staining

Antibody	Dilution	Supplier	Cat#	RRID
Rhodopsin (mouse)	1:1000	Millipore, Burlington, MA, USA	MAB5356	AB_2178961
Red/green opsin (rabbit)	1:1000	Millipore	AB5405	AB_177456
Rat anti-glial fibrillary acidic protein	1:1000	Thermo Fisher Scientific, Waltham, MA, USA	13-0300	AB_2748894
Donkey anti-mouse IgG conjugated to Alexa Fluor-647	1:1000	Invitrogen, Waltham, MA, USA	A31573	AB_2536183
Donkey anti-rabbit IgG conjugated to Alexa Fluor-488	1:1000	Invitrogen	A21206	AB_2535792
Donkey anti-rat IgG conjugated to Alexa Fluor-488	1:1000	Invitrogen	A21208	AB_141709

Immunofluorescence-labeled tissues were examined using a confocal microscope (LSM700, Zeiss, Oberkochen, Germany) or a fluorescence microscope (Zeiss). To assess photoreceptor viability, the thickness and number of cell layers in the outer nuclear layer (ONL), which houses photoreceptor cell bodies, were measured according to a previously described method. Because of ONL irregularities resulting from MNU-induced degeneration, measurements were obtained at defined distances of 400, 800, 1200, and 1600 µm from the center of the optic nerve on both sides. To evaluate photoreceptor structure, the length or thickness of the outer segments (OSs) of cones (labeled with opsin) and rods (labeled with rhodopsin) was measured at a distance of 800 µm from the optic nerve center. In the same region, the fluorescence intensity and total fluorescent area of glial fibrillary acidic protein (GFAP) were quantified as indicators of Müller glial cell reactivity. All retinal sections from each experimental group were subjected to identical immunostaining, imaging, and analysis procedures. Measurements were conducted using Image-Pro Plus software (Media Cybernetics, Rockville, MD, USA). For each mouse, values from three retinal sections were averaged to generate a single data point; group means were calculated from these individual values.

### Western blotting

To examine protein expression in the retina, western blot analysis was conducted according to a previously described protocol. Retinas were promptly excised from the eyecups and immediately placed in ice-cold lysis buffer. Tissues were lysed at 4°C for 30 minutes. For electrophoresis, at least 30 µg of retinal protein per lane were loaded onto a 4%–12% polyacrylamide gradient gel (Smart-Lifesciences Biotechnology, Changzhou, China). After electrophoresis, proteins were transferred onto a polyvinylidene difluoride membrane (Millipore, Billerica, MA, USA).

Membranes were blocked with a rapid-blocking solution (Epizyme, Shanghai, China, Cat# PS108) for 30 minutes. They were then incubated overnight at 4°C with primary antibodies under gentle agitation. Next, membranes were washed, then incubated with the secondary antibodies at 25°C for 2 hours. After three additional washes, the blots were visualized using a chemiluminescence detection system (Bio-Rad, Hercules, CA, USA). Semi-quantitative analyses were conducted using ImageJ software (Version 1.8.0.112, National Institutes of Health, Bethesda, MD, USA). Details of the primary and secondary antibodies are listed in **Table 2**.

**Table 2:** Antibodies used for western blotting

Antibody	Dilution	Supplier	Cat#	RRID
Rabbit anti-Bax	1:1000	Cell Signaling Technology, Danvers, MA, USA	14796S	AB_2716251
Rabbit anti-Bcl-2	1:1000	HUABIO	ET1702-53	AB_3070315
Rabbit anti-p44/42 MAPK (Erk1/2)	1:1000	Cell Signaling Technology	9102S	AB_330744
Rabbit anti-phospho-p44/42 Rabbit anti-MAPK (Erk1/2)	1:1000	Cell Signaling Technology	4370S	AB_2315112
Rabbit anti-GAPDH	1:3000	Sangon Biotech, Shanghai, China	D110016	AB-2904600
HRP-conjugated goat anti-rabbit IgG(H+L)	1:5000	Proteintech, Rosemont, IL, USA	SA00001-2	AB_2722564

Erk: Extracelluar regulated protein kinases; GAPDH: glyceraldehyde-3-phosphate dehydrogenase; MAPK: mitogen-activated protein kinase.

### Statistical analysis

No statistical methods were used to predetermine sample sizes; however, the sample sizes were consistent with those reported in previous studies (Xiang et al., 2018; Zhang et al., 2022a). Statistical analyses were performed using GraphPad Prism version 9.0 (GraphPad Software, San Diego, CA, USA, www.graphpad.com). Data are presented as mean ± standard error of the mean. One-way analysis of variance followed by Tukey’s *post hoc* test was used for comparisons among multiple groups. *P*-values < 0.05 were considered statistically significant.

## Results

### *Lycium barbarum* glycopeptide and luteolin mixture improves visual function in N-nitroso-N-methylurea-injured mice

To evaluate the effects of the LbGP and luteolin mixture on visual function, we first assessed the innate preference of mice for darkness using a dark–light transition box (**Figure 1B**). Because of their nocturnal behavior, control mice with normal vision typically spent more time in the dark compartment than in the illuminated one. In contrast, MNU-treated mice, which exhibit photoreceptor degeneration, showed impaired light perception and distributed their time more evenly between the two compartments (representative movement trace shown in **Figure 1C**). Treatment with the LbGP and luteolin mixture significantly increased time spent in the dark compartment by 23.2% relative to the MNU group (*P* < 0.01; **Figure 1D**).

Visual acuity was subsequently measured using the optomotor test (**Figure 1E**). A substantial reduction in visual acuity was observed in MNU-injured mice relative to controls. However, treatment with the LbGP and luteolin mixture greatly improved visual performance, leading to an increase of 115% compared with the findings in the MNU group (*P* < 0.001; **Figure 1F**). These results indicate that the LbGP and luteolin mixture improved visual function in MNU-injured mice.

### *Lycium barbarum* glycopeptide and luteolin mixture improves retinal light response in N-nitroso-N-methylurea-injured mice

**Figure 2:**
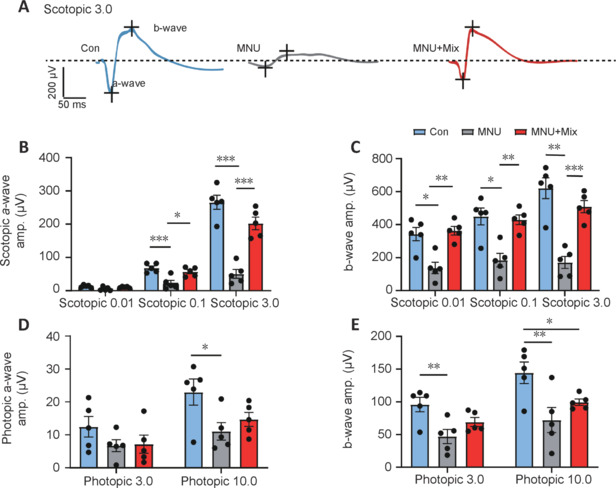
LbGP and luteolin mixture improves retinal light response in MNU-injured mice. (A) Representative ERG waveforms from each group in response to flashes at a scotopic intensity of 3.0 cd∙s/m^2^. (B, C) Peak amplitudes of the a-wave (B) and b-wave (C) under scotopic conditions at various flash intensities (*n* = 5 per group). (D, E) Peak amplitudes of the a-wave (D) and b-wave (E) under photopic conditions at different flash intensities (*n* = 5 per group). Data are expressed as mean ± SEM; *P < 0.05, **P < 0.01, ***P < 0.001 (one-way analysis of variance with Tukey’s *post hoc* test). Group: Con, Control; MNU, MNU-induced retinal injury in mice; MNU+Mix, MNU-injured mice treated with a mixture of LbGP and luteolin. amp: Amplitude; LbGP: *Lycium barbarum* glycopeptide; MNU: N-nitroso-N-methylurea.

Next, ERG was used to assess retinal neuronal function. ERG is a non-invasive electrophysiological technique that measures retinal responses to light stimuli; a-wave and b-wave components represent the activity of photoreceptors and downstream bipolar cells, respectively (Zhang et al., 2022b). Under dark-adapted conditions (Scotopic 3.0), control mice exhibited robust responses to light flashes (**Figure 2A**). In contrast, MNU-treated mice showed significantly reduced responses. Treatment with the LbGP and luteolin mixture partially restored the light response (**Figure 2A**).

Under scotopic conditions, both a-wave and b-wave amplitudes were significantly increased after treatment with the LbGP and luteolin mixture relative to the MNU group, particularly at higher flash intensities (Scotopic 3.0: a-wave, *P* = 0.0007; b-wave, *P* = 0.0005; **Figure 2B** and **C**). However, the improvement was not statistically significant under light-adapted conditions (Photopic 3.0: a-wave, *P* = 0.99; b-wave, *P* = 0.44; Photopic 10.0: a-wave, *P* = 0.64; b-wave, *P* = 0.29; **Figure 2D** and **E**). These findings suggest that the LbGP and luteolin mixture enhances the retinal light response of rod pathways in MNU-injured mice.

### *Lycium barbarum* glycopeptide and luteolin mixture protects retinal structures in N-nitroso-N-methylurea-injured mice

**Figure 3:**
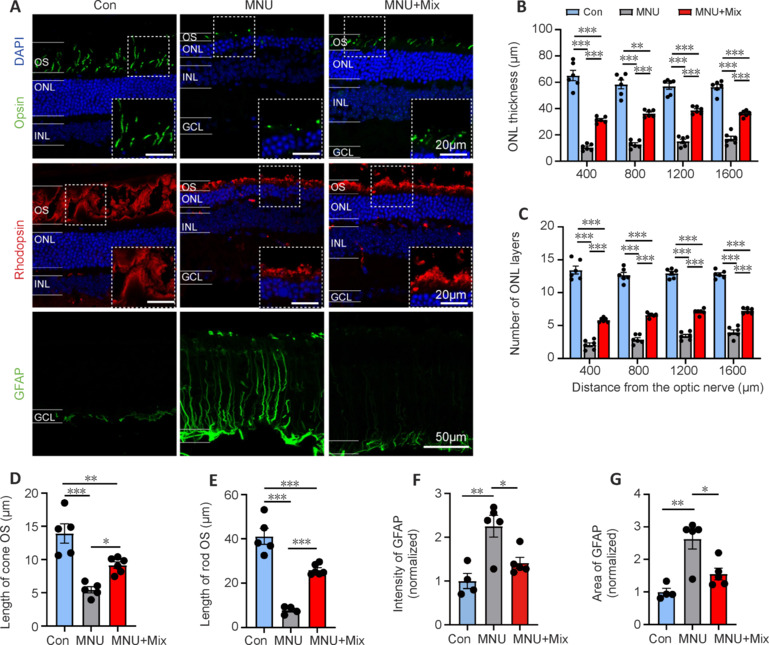
LbGP and luteolin mixture protects retinal structures in MNU-injured mice. (A) Representative images of retinal sections from the intermediate region of the retina. DAPI (blue) labels nuclei; opsin (Alexa Fluor™ 488, green) labels cone OS; rhodopsin (Alexa Fluor^TM^ 647, red) labels rod OS; GFAP (Alexa Fluor^TM^ 488, green) labels Müller glial cells. (B, C) Quantification of ONL thickness (B) and number of cell layers (C). Retinal sections from each group were analyzed from central to peripheral regions, with measurements taken at 400 µm, 800 µm, 1200 µm, and 1600 µm from the optic nerve center (*n* = 6 per group). (D, E) Length of cone OS (D) and rod OS (E) in each group. Cone OS: *n* = 4 or 6; rod OS: *n* = 5 or 6. (F, G) Fluorescence intensity (F) and area (G) of GFAP-positive regions, normalized to control values (*n* = 5 per group). Treatment with the LbGP and luteolin mixture improved photoreceptor survival and structure while suppressing Müller cell activation in MNU-injured mice. Data expressed as mean ± SEM; *P < 0.05, **P < 0.01, ***P < 0.001 (one-way analysis of variance with Tukey’s *post hoc* test). Group: Con, Control; MNU, MNU-induced retinal injury in mice; MNU + Mix, MNU-injured mice treated with a mixture of LbGP and luteolin. DAPI: 4′,6-Diamidino-2-phenylindole; GCL: ganglion cell layer; GFAP: glial fibrillary acidic protein; INL: inner nuclei layer; LbGP: *Lycium barbarum* glycopeptide; MNU: N-nitroso-N-methylurea; ONL: outer nuclear layer; OS: outer segments.

After we had confirmed functional improvement, we evaluated the effect of the mixture on retinal morphology. **Figure 3A** presents representative images of central retinal sections from each experimental group, stained with opsin, rhodopsin, and GFAP to label cone OSs, rod OSs, and Müller cell processes, respectively. In the MNU group, the ONL appeared substantially thinner and showed a reduced number of cell layers compared with the findings in the control group. In contrast, retinas from mice treated with the LbGP and luteolin mixture exhibited robust preservation of ONL thickness and cell layer count. Quantitative analysis confirmed that the mixture significantly increased ONL thickness and the number of cell layers in MNU-injured retinas (Mixture *vs.* MNU; **Figure 3B** and **C**). At a distance of 800 µm from the optic nerve, ONL thickness and cell layer count in the MNU group declined to 21.9% and 22.8% of control values, respectively (*P* < 0.001). Treatment with the LbGP and luteolin mixture restored ONL thickness and cell layer count to 62.2% and 52.0% of control levels, respectively (*P* < 0.001).

The morphological features of cone and rod photoreceptors were quantified by measuring the lengths of their OSs at a distance of 800 µm from the optic nerve. As illustrated in **Figure 3A**, cone OSs in the control group appeared as long, densely packed strips, whereas those in the MNU group were sparse and punctate. After oral administration of the LbGP and luteolin mixture, cone OSs regained elongated, strip-like structures. Similarly, rhodopsin staining in the control group formed a large, intensely stained sheet; in the MNU group, it appeared as small, patchy regions. After treatment with the mixture, rhodopsin staining resembled a continuous mountain-peak-like sheet. In the middle retinal zone (800 µm from the optic nerve), OS lengths in the MNU group were reduced to 39.1% (cones) and 19.0% (rods) of control values (*P* < 0.001). Treatment with the mixture restored cone and rod OS lengths to 65.8% and 63.2% of control values, respectively (cones: *P* < 0.05; rods: *P* < 0.001; **Figure 3D** and **E**). These findings indicate that the LbGP and luteolin mixture delayed cone and rod degeneration in MNU-injured mice.

Upregulation of GFAP in Müller cell processes is a hallmark of reactive gliosis and serves as an indicator of retinal inflammation (Bringmann and Wiedemann, 2012). In the control group, Müller glial cells did not exhibit GFAP activation, and GFAP expression was limited to astrocytes in the ganglion cell layer. In contrast, the MNU group showed robust GFAP upregulation in Müller cell processes extending from the ganglion cell layer to the ONL, indicating extensive Müller glial activation. Treatment with the LbGP and luteolin mixture reduced GFAP staining in Müller cells (**Figure 3A**). Quantitative analysis revealed a 2.3-fold increase in GFAP fluorescence intensity and a 2.6-fold increase in GFAP expression area in the MNU group compared with the findings in the control group (*P* < 0.01). After treatment, these values decreased to 1.4-fold and 1.6-fold of control levels, respectively (*P* < 0.05; **Figure 3F** and **G**). In summary, the LbGP and luteolin mixture preserved retinal structure in MNU-injured mice by improving retinal morphology, delaying cone and rod degeneration, and reducing GFAP expression in Müller glial cells.

### *Lycium barbarum* glycopeptide and luteolin mixture inhibits activation of the ERK–Bax/Bcl-2 pathway in N-nitroso-N-methylurea-injured mice

**Figure 4:**
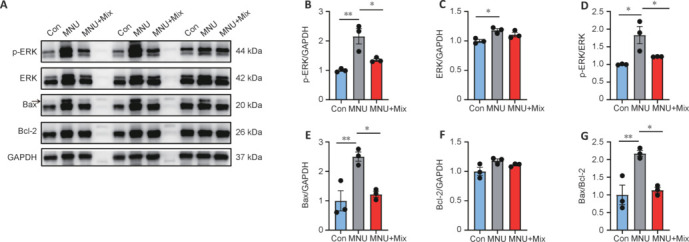
LbGP and luteolin mixture inhibits activation of the ERK–Bax/Bcl-2 pathway in MNU-injured mice. (A) Western blot showing protein expression levels of ERK, phosphorylated ERK (p-ERK), Bax, and Bcl-2. GAPDH was used as a loading control to confirm equal protein loading across samples. (B–D) Quantitative analysis of p-ERK/GAPDH, ERK/GAPDH, and p-ERK/ERK ratios. (E–G) Quantification of Bax/GAPDH, Bcl-2/GAPDH, and Bax/Bcl-2 ratios. *n* = 3 per group. Data are expressed as mean ± SEM. The data were derived from three independent experiments. *P < 0.05, **P < 0.01 (one-way analysis of variance with Tukey’s *post hoc* test). Group: Con, Control; MNU, MNU-induced retinal injury in mice; MNU + Mix, MNU-injured mice treated with a mixture of LbGP and luteolin. LbGP: *Lycium barbarum* glycopeptide; MNU: N-nitroso-N-methylurea.

After we had established the protective effect of the mixture, we explored the underlying signaling pathways involved. We first examined inflammatory signaling by focusing on extracellular signal-regulated kinase (ERK), a key component of the mitogen-activated protein kinase pathway that responds to inflammatory stimuli (Yao et al., 2017). Relative to the control group, the MNU group displayed elevated expression of phosphorylated ERK (p-ERK), as well as total ERK. Treatment with the mixture primarily reduced p-ERK levels, with minimal effect on total ERK expression (**Figure 4A–C**). While the p-ERK/ERK ratio in the MNU group was 1.83-fold of that in the control group (*P* < 0.05), the mixture-treated group reduced it to 1.22-fold of control (*P* < 0.05). Consequently, the p-ERK to total ERK ratio, a marker of ERK pathway activation, was significantly decreased in the mixture-treated group. These findings indicate that the LbGP and luteolin mixture effectively inhibited ERK pathway activation in MNU-injured retinas (**Figure 4D**).

Given that ERK activation can influence the expression of Bcl-2 family proteins, we investigated the levels of Bcl-2, an anti-apoptotic protein, and Bax, a pro-apoptotic protein (Lavoie et al., 2020). Western blot analysis revealed increased expression of both Bax and Bcl-2 in the MNU group. Treatment with the mixture led to a reduction in Bax levels, whereas Bcl-2 levels remained relatively stable (**Figure 4A**, **E**, and **F**). The Bax/Bcl-2 ratio, an indicator of apoptosis pathway activation, was significantly reduced in the mixture-treated group relative to the MNU group (*P* < 0.05; **Figure 4G**). These results suggest that the LbGP and luteolin mixture inhibited apoptosis signaling in MNU-injured retinas.

### *Lycium barbarum* glycopeptide and luteolin mixture outperforms single agents in protection of N-nitroso-N-methylurea-injured photoreceptors

**Figure 5:**
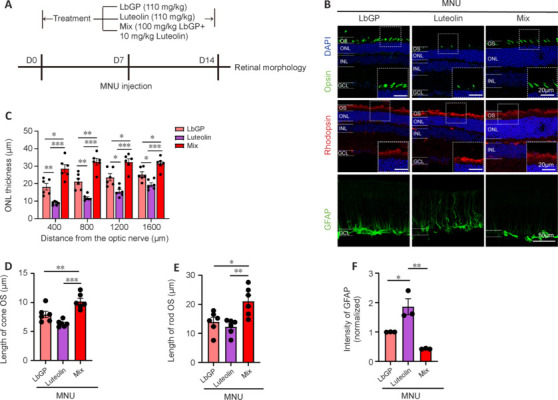
LbGP and luteolin mixture outperforms single agents in protecting MNU-injured photoreceptors. (A) Experimental design. (B) Representative images of retinal sections from the intermediate region. DAPI (blue) labels nuclei; opsin (Alexa Fluor^TM^ 488, green) labels cone outer segments (OS); rhodopsin (Alexa Fluor^TM^ 647, red) labels rod OS; GFAP (Alexa Fluor^TM^ 488, green) labels Müller glial cells. Scale bars: 50 µm. (C) Quantification of ONL thickness. (D, E) Quantification of cone OS length (D) and rod OS length (E) in each group. (F) Fluorescence intensity of GFAP-positive regions, normalized to control values. Compared with LbGP or luteolin monotherapy at 110 mg/kg, the combination of LbGP and luteolin more effectively preserved photoreceptor survival and structure, while suppressing Müller cell activation. Data are expressed as mean ± SEM; *P < 0.05, **P < 0.01, ***P < 0.001 (two-way or one-way analysis of variance with Tukey’s *post hoc* test). DAPI: 4′,6-Diamidino-2-phenylindole; GCL: ganglion cell layer; INL: inner nuclear layer; LbGP: *Lycium barbarum* glycopeptide; MNU: N-nitroso-N-methylurea; ONL: outer nuclei layer.

After we had clarified the protective effect of the LbGP and luteolin mixture on the MNU-injured retina, along with its inhibitory impacts on the ERK and Bcl-2 signaling pathways, we compared the efficacy of the combined treatment with that of each individual agent in protecting retinal photoreceptors. **Figure 5A** provides a detailed overview of the experiment. The LbGP and luteolin mixture demonstrated superior efficacy across multiple structural indicators, including increased ONL thickness, elongation of cone and rod OSs, and suppression of GFAP expression (**Figure 5B–F**). At 400 µm from the optic nerve, ONL thickness in the mixture-treated group was 57.4% greater than that in the LbGP group (*P* < 0.05) and 226.7% greater than that in the luteolin group (*P* < 0.001). Cone OS length increased by 28.4% compared with that with LbGP alone (*P* < 0.01) and by 59.7% compared with that with luteolin alone (*P* < 0.001). GFAP expression in Müller cells was reduced to 42.7% of the LbGP group’s level (*P* < 0.05) and 22.9% of the luteolin group’s level after mixture treatment (*P* < 0.05). These findings confirmed that the LbGP and luteolin mixture provides more robust protection against photoreceptor degeneration than either agent alone.

## Discussion

Our results demonstrate that LbGP, in combination with luteolin, attenuates MNU-induced degeneration of retinal photoreceptors and improves both visual function and retinal light responses. This protective effect is possibly mediated by inhibition of the ERK and Bax/Bcl-2 signaling pathways. Furthermore, the combination exhibits a synergistic effect, offering greater retinal protection than either component administered individually.

This study builds upon our previous work (Kong et al., 2024), which showed that LbGP alone improves the structural integrity and viability of photoreceptors in mice with MNU-induced degeneration, while also enhancing retinal light responses and visual behavior. In prior experiments (Kong et al., 2024), the group receiving treatment with LbGP monotherapy at a dose of 100 mg/kg showed increased ONL thickness (at 800 μm from the optic nerve) and time spent in the dark box by 65.49% and 13.93%, respectively, compared with the MNU-injured group. In contrast, combination therapy with LbGP and luteolin led to increases of 132.47% and 23%, respectively. Moreover, LbGP alone did not significantly suppress GFAP activation, resulting in only a 7.55% reduction relative to the MNU group, whereas the combined therapy decreased GFAP activation by 37.15%. These results indicate that luteolin addition enhances the therapeutic efficacy of LbGP in MNU-injured retinas.

The superior effect of the combination may result from a synergistic interaction between LbGP and luteolin. Previous studies have shown that luteolin at a dose of 100 mg/kg can rescue photoreceptors in rd10 mice, a transgenic model of RP, and effectively inhibit activation of both Müller glia and microglia (Liu et al., 2021b). In the present study, we administered luteolin at a lower dose of 10 mg/kg with the goal of reducing inflammation (Zhang et al., 2021). The mixture effectively suppressed MNU-induced retinal inflammation. However, inhibition of Müller cell activation at this lower dose of luteolin was less pronounced than with LbGP alone, suggesting that the observed synergy is not solely driven by the anti-inflammatory effects of luteolin; its strong antioxidant properties may instead explain the enhanced therapeutic outcome.

In addition to its superior protective effect, combination therapy offers another advantage over monotherapy: the potential to delay or prevent the development of drug resistance. Although LbGP is effective in treating MNU-induced retinal degeneration, prolonged use may cause reduced efficacy due to the emergence of resistance, a common issue in long-term pharmacological treatment. For example, extended administration of berberine induces bacterial resistance through mechanisms such as efflux pump upregulation and structural changes in target enzymes (Tong et al., 2021). Notably, the combination of berberine with curcumin enhances antibacterial efficacy, reduces resistance development, and exerts strong anti-inflammatory effects to promote wound healing (Kandaswamy et al., 2024). Given the potential for similar resistance to develop with LbGP, combination therapy may serve as a strategy to mitigate this risk and sustain long-term therapeutic benefits.

MNU-induced photoreceptor loss has been attributed to the inhibition of DNA methylation adduct formation in photoreceptor nuclei through alkylation (Ogino et al., 1993). In addition to causing DNA damage, MNU induces apoptosis (Tsubura et al., 2010), excessive oxidative stress, mitochondrial loss (Chen et al., 2014), activation of microglia, and inflammation (Zhang et al., 2018). In one study, nicotinamide successfully mitigated photoreceptor loss in MNU-injured rodent models, although levels of 7-methyldeoxyguanosine—a marker of DNA alkylation—remained elevated (Kiuchi et al., 2002). In contrast, plant extracts have been reported to treat MNU-injured retinas effectively by targeting inflammation, oxidative stress, or apoptosis (Park et al., 2021; Qi et al., 2023b). These findings suggest that, similar to other plant-based treatments, the LbGP and luteolin mixture acts downstream of DNA alkylation by inhibiting inflammation and apoptosis, rather than directly facilitating DNA repair.

Beyond its anti-inflammatory effect, our results indicate that the LbGP and luteolin mixture also inhibits apoptosis through dual modulation of the ERK and Bax/Bcl-2 signaling pathways. It is known that ERK hyperactivation drives photoreceptor apoptosis (Garg and Chang, 2003; Shen et al., 2010). Our current study found that LbGP inhitibite the ERK hyepractivaiton as well as Bax. The mixture initially reduced the p-ERK/ERK ratio, suppressing hyperactivation of the ERK pathway. This pathway plays a critical role in cell proliferation, differentiation, survival, and stress responses (Sun et al., 2015). Because excessive ERK activation can promote cellular stress and apoptosis, its suppression may help restore intracellular homeostasis and prevent apoptotic signaling. ERK activity also influences the expression of Bcl-2 family proteins during MNU-induced injury (Sugano et al., 2019); thus, inhibition of the ERK pathway may further modulate these apoptotic regulators. Western blot analysis confirmed a significant reduction in the Bax/Bcl-2 ratio after treatment with the mixture, indicating a decreased propensity for apoptosis. Concomitant inhibition of ERK pathway activation and modulation of Bax/Bcl-2 imbalance highlights the therapeutic potential of the LbGP and luteolin mixture in preserving photoreceptor cells and maintaining retinal function.

The study had some limitations. First, the small sample size (*n* = 3 per group) might have limited the power to detect significant effects. Second, only the short-term protective effects of the mixture after MNU induction were assessed; the long-term efficacy remains unclear. Third, the experiment included only male mice; future studies should incorporate female mice to evaluate potential sex-based differences. Fourth, the translational relevance to human retinal disease remains uncertain. Fifth, the study relied solely on a single chemical injury model. Inherited photoreceptor degeneration models such as rd10 mice would extend its application. Indeed, in a pilot study, we administered LbGP alone at 100 mg/kg to rd10 mice by oral gavage from postnatal day 12 (P12), prior to the onset of rod photoreceptor death (beginning at P17), until P25, when rod apoptosis peaks. However, no significant protective effect was observed. Before directly comparing the efficacy of the mixture in MNU and rd10 models, it may be necessary to optimize the timing (e.g., earlier than P12) and dosage of LbGP monotherapy to achieve measurable protection in rd10 mice. To address these limitations, future studies will employ genetically modified mice instead of chemical injury models, increase the sample size, and optimize drug concentrations for treatment efficacy. We will also conduct long-term safety assessments to provide a more comprehensive evaluation of the treatment’s potential. Nevertheless, differences in underlying mechanisms of photoreceptor degeneration between MNU-induced and rd10 models may limit the generalizability of these findings.

In conclusion, this study demonstrated that the LbGP and luteolin mixture confers protective effects against MNU-induced photoreceptor degeneration, potentially through dual regulation of the ERK and Bax/Bcl-2 signaling pathways. This combination may represent a promising therapeutic strategy for the management of retinal degenerative conditions.

## Data Availability

*No additional data are available*.
